# Identification of PLA2G7 as a novel biomarker of diffuse large B cell lymphoma

**DOI:** 10.1186/s12885-021-08660-4

**Published:** 2021-08-17

**Authors:** Weili Zheng, Qiaochu Lin, Mohammed Awal Issah, Ziyuan Liao, Jianzhen Shen

**Affiliations:** 1 Fujian Institute of Hematology, Fujian Medical Center of Hematology, Fujian Provincial Key Laboratory on Hematology; Fujian Medical University Union Hospital, Fuzhou, China; 2 Meng Chao Hepatobiliary Hospital Affiliated to Fujian Medical University, Fuzhou, China

**Keywords:** Diffuse large B-cell lymphoma, Receiver operating characteristic curve, Weighted gene co-expression network analysis, Tumor microenvironment, PLA2G7

## Abstract

**Background:**

Diffuse large B-cell lymphoma is the most common form of non-Hodgkin lymphoma globally, and patients with relapsed or refractory DLBCL typically experience poor long-term outcomes.

**Methods:**

Differentially expressed genes associated with DLBCL were identified using two GEO datasets in an effort to detect novel diagnostic or prognostic biomarkers of this cancer type, after which receiver operating characteristic curve analyses were conducted. Genes associated with DLBCL patient prognosis were additionally identified via WCGNA analyses of the TCGA database. The expression of PLA2G7 in DLBCL patient clinical samples was further assessed, and the functional role of this gene in DLBCL was assessed through in vitro and bioinformatics analyses.

**Results:**

DLBCL-related DEGs were found to be most closely associated with immune responses, cell proliferation, and angiogenesis. WCGNA analyses revealed that PLA2G7 exhibited prognostic value in DLBCL patients, and the upregulation of this gene in DLBCL patient samples was subsequently validated. PLA2G7 was also found to be closely linked to tumor microenvironmental composition such that DLBCL patients expressing higher levels of this gene exhibited high local monocyte and gamma delta T cell levels. In vitro experiments also revealed that knocking down PLA2G7 expression was sufficient to impair the migration and proliferation of DLBCL cells while promoting their apoptotic death. Furthmore, the specific inhibitor of PLA2G7, darapladib, could noticeably restrained the DLBCL cell viability and induced apoptosis.

**Conclusions:**

PLA2G7 may represent an important diagnostic, prognostic, or therapeutic biomarker in patients with DLBCL.

**Supplementary Information:**

The online version contains supplementary material available at 10.1186/s12885-021-08660-4.

## Introduction

Lymphomas are a very prevalent form of cancer that can be classified into Hodgkin and non-Hodgkin lymphoma subtypes (HL and NHL, respectively). Diffuse large B-cell lymphoma (DLBCL) is the most common NHL subtype globally, accounting for 30–40% of overall NHL cases [1]. DLBCL is a highly heterogeneous and aggressive disease that can exhibit highly varied outcomes in affected patients. Treatment of DLBCL patients with rituximab and cyclophosphamide-doxorubicin-vincristine-prednisone chemotherapy (R-CHOP) has led to rising long-term patient survival and a 50–75% 5-year survival rate for patients with this disease [2]. However, roughly 40% of patients ultimately suffer from relapsed or refractory disease [3]. The genetic and molecular etiology of DLBCL is also highly complex and some studies have focused on the identification of genetic drivers and their functional roles in DLBCL to determine novel therapeutic targets and/or diagnostic or prognostic biomarkers to promote the disease’s diagnosis and treatment [4, 5].

High-throughput and next-generation sequencing (NGS) technologies have enabled many biomarker discovery studies in DLBCL samples to date [6, 7]. Findings from the majority of these studies, however, have yet to be validated or implemented in clinical settings. Many of these prior studies have also been limited by factors such as tissue heterogeneity, small sample sizes, and single-cohort approaches, resulting in inconsistent results [8]. One approach to overcoming these limitations is the re-analysis of multiple independent previously published NGS datasets in order to more reliably identify potential cancer-specific biomarkers [9, 10].

Herein, we sought to identify novel DLBCL diagnostic, prognostic, or therapeutic biomarkers via an integrative bioinformatics approach. Through this strategy, we identified phospholipase A2 group VII (PLA2G7) as a novel biomarker of interest associated with this cancer type. PLA2G7 has previously been reported to control a range of oncogenic processes through mechanisms associated with metabolic alterations [11]. PLA2G7 has also been identified as a prognostic biomarker in patients with prostate cancer (PCa) [12] and melanoma [13]. Together, our data suggest that PLA2G7 may function as a driver of the proliferation, migration, and survival of tumor cells and a regulator of immune cell infiltration within the DLBCL tumor microenvironment. PLA2G7 may therefore represent a viable therapeutic target in DLBCL patients.

## Materials and methods

### Ethics statement and clinical specimens

The study was approved by the Ethics Committee of Fujian Medical University Union Hospital. All experiments were performed according to the relevant regulations and written informed consent was obtained from patients. A total of 18 DLBCL tissues, 11 lymphadenitis tissues and 53 DLBCL peripheral blood samples were obtained from Fujian Medical University Union Hospital. Tissue samples were frozen in liquid nitrogen until further analysis. DLBCL cases only were confirmed by pathological examination of lymph node biopsy or lymphadenectomy according to the 2017 WHO Classification of Lymphoid Neoplasms. Patients samples used for this study had not received chemotherapy or radiotherapy before surgery.

### DEG identification

The GSE32018 and GSE56315 datasets from the Gene Expression Omnibus (GEO) database (http://www.ncbi.nlm.nih.gov/geo/) were downloaded. Raw CEL files were background corrected, subjected to z-score transformation and quantile normalization, and subjected to further analysis. The GSE32018 dataset [14] contained 22 DLBCL tumor samples and 7 normal lymph node tissue samples and was prepared using the GPL6480 platform. The GSE56315 dataset [15] contained 55 DLBCL tissue samples and 33 normal tonsil tissues and was prepared using the GPL570 platform.

In addition, gene expression profiles and clinical data pertaining to 48 DLBCL patients were downloaded from The Cancer Genome Atlas (TCGA) database (https://portal.gdc.cancer.gov/). The ESTIMATE algorithm was used to calculate stromal and immune scores [16].

Genes that were differentially expressed between DLBCL and control tissues were identified with the R ‘limma’ package using the following cutoff criteria: log2 FoldChange (FC) > 1 and adjusted *P*-value < 0.05. The RobustRankAggreg (RRA) R package was then used to integrate the DEGs from these two datasets. RRA utilizes a probabilistic model to aggregate and monitor the genes that are ranked consistently better than expected under the null hypothesis of uncorrelated inputs, and defines a significance score for each gene [17]. Cutoff criteria: score < 0.05 was applied.

### Functional enrichment analysis

Identified DEGs were subjected to Gene Ontology (GO) and Kyoto Encyclopedia of Genes and Genomes (KEGG) pathway enrichment analyses [18–20] using the DAVID database (https://david.ncifcrf.gov/summary.jsp), with *P <* 0.05 as the threshold of statistical significance.

### Weighted gene co-expression network analysis (WGCNA)

Co-expression analyses were conducted using the WGCNA R package using TCGA data corresponding to 48 DLBCL patients for whom detailed survival data were available. A weighted adjacency matrix was constructed by determining Pearson’s correlation coefficients for individual pairs of genes. An appropriate soft power threshold was then selected to yield standardized scale-free networks, after which the resultant matrix was subjected to transformation into a topological overlap matrix (TOM). In addition, topological overlap dissimilarity (1-TOM) was utilized as input for hierarchical clustering analyses. Gene dendrogram and module identification were constructed with a dynamic tree cut. Any modules with a size < 30 were then deleted. Modules that had a dissimilarity of < 0.25 were merged, after which relationships between clinical traits and module eigengenes were assessed.

### Cell culture and transfection

Human DB and SU-DHL-2 DLBCL cells were obtained from Procell Life Science and Technology and were grown in RPMI-1640 (Invitrogen, CA, USA) containing 10% fetal bovine serum (Gibco, CA, USA) at 37 °C in a 5% CO2 humidified incubator. Cells were transfected with Negative Control (NC) and PLA2G7 siRNAs using Lipofectamine 3000 (Invitrogen). The siRNA sequences were as follows: PLA2G7-si1: CCUGUUGCCCAUAUGAAAUTT,

AUGGUUAAUGUUUGCAGGCAT; PLA2G7-si2: CCUGGAUGCAUGGAUGUUUTT,

AAACAUCCAUGCAUCCAGGGC.

### Quantitative real-time PCR

Trizol (Invitrogen) was utilized to extract RNA from cells, after which a cDNA synthesis kit (Thermo Fisher Scientific, USA) was used based on provided directions to prepared cDNA. The expression levels were detected by QRT-PCR analysis with FastStart Universal SYBR-Green Master (Roche), an ABI7500 sequence detector (Applied Biosystems, Foster City, CA, USA) and calculated by 2 − ΔΔCt method. β-actin expression was used for normalization purposes, and primers used in this study were as follows: PLA2G7, Forward (F): 5′-GAACACACTGGCTTATGGGC-3′, Reverse (R): 5′-GAGATGCCAGGTCAATGCCA-3′.

### Colony formation and migration assays

For colony formation assays, 500 cells were added per well in methylcellulose-based media. Plates were then cultured for 2 weeks, after which methanol was used to fix colonies which were subsequently stained with 0.5% crystal violet. All colonies containing > 50 cells were then counted via microscopy.

For migration assays, 24-well transwell chambers (8 μm aperture, BD Biosciences) were utilized. DLBCL cells were suspended in FBS-free media and were added to the upper well of these chambers, whereas cells in media supplemented with 10% FBS were added into the lower chamber. Following a 48 h incubation, cells that had not migrated to the lower chamber were removed with a cotton swab. Migrated cells were then fixed and stained using 0.5% crystal violet and imaged via microscopy. In total, five fields of view per sample were analyzed at random. ImageJ was utilized to quantify the number of migrated cells per well.

### Apoptosis assay

A PE Annexin V Apoptosis detection kit (BD Pharmingen, USA) was utilized at 48 h post-transfection to analyze cells. Briefly, cells were stained using Annexin V-PE and 7-AAD for 15 min at room temperature. A BD Accuri C6 flow cytometer was then used to analyze samples, after which FlowJo was used for data analysis.

### CCK-8 assay

Cells were seeded into 96-well plates and incubated in different concentrations of darapladib for 72 h, then CCK-8 dye solution was added to each well and incubated at 37 °C for 2 h. The absorbance was measured at 450 nm using a microplate reader (BioTek, USA).

### Statistical analysis

ROC (receiver operating characteristic) curve was conducted to analyze the effectiveness of target gene expressions between tumor and healthy samples. Area under the ROC curve (AUC) values were calculated using SPSS 20.0 (SPSS Inc., IL, U.S.A.) and were used to assess the sensitivity and specificity of individual DEGs as diagnostic biomarkers between DLBCL and normal tissues.

Patients were separated into two groups based upon PLA2G7 expression levels, after which two-tailed chi-squared tests were utilized to compare clinicopathological characteristics between the PLA2G7-high and –low patient groups. In addition, comparisons of PLA2G7 expression levels were made via Student’s t-tests and one-way ANOVAs using GraphPad Prism 8. Kaplan-Meier survival curves were drawn to show the relationship between expression of PLA2G7 and overall survival (OS) of patients, which was tested by the log-rank test. *P* < 0.05 was the significance threshold for this analysis.

## Results

### Integrative DEG identification

**Fig. 1:**
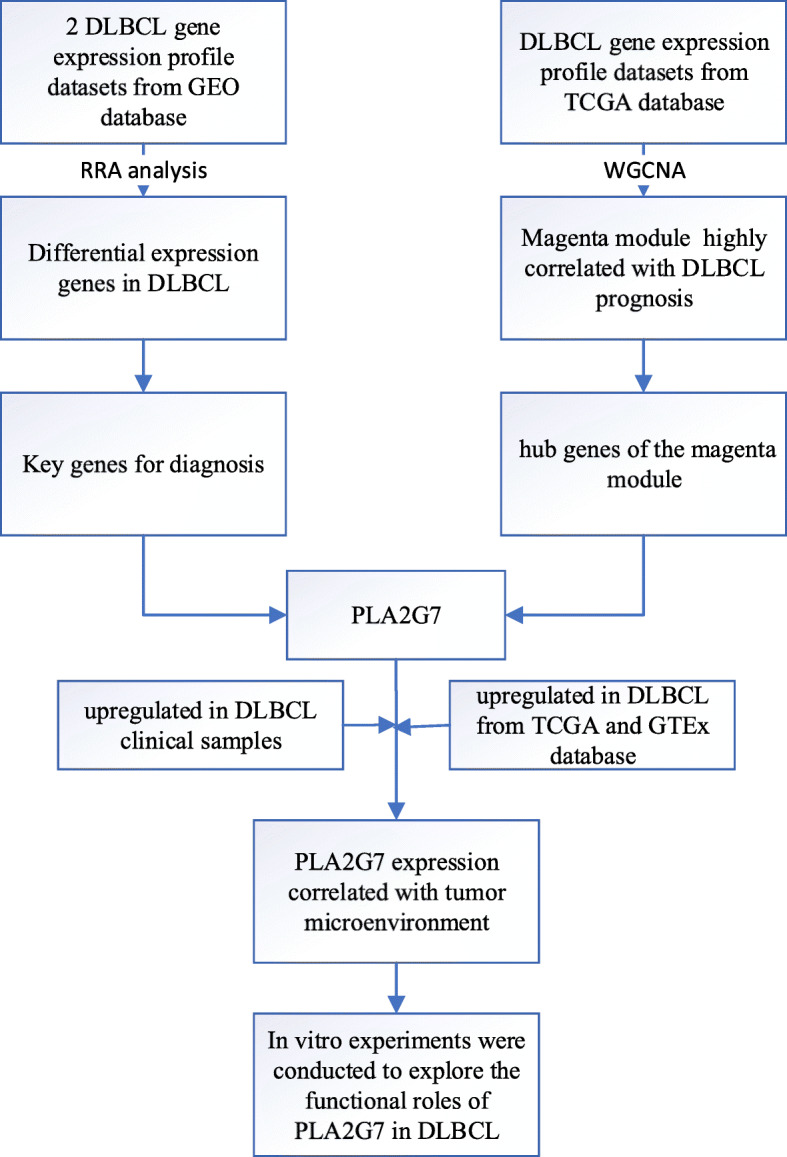
Study overview

**Fig. 2:**
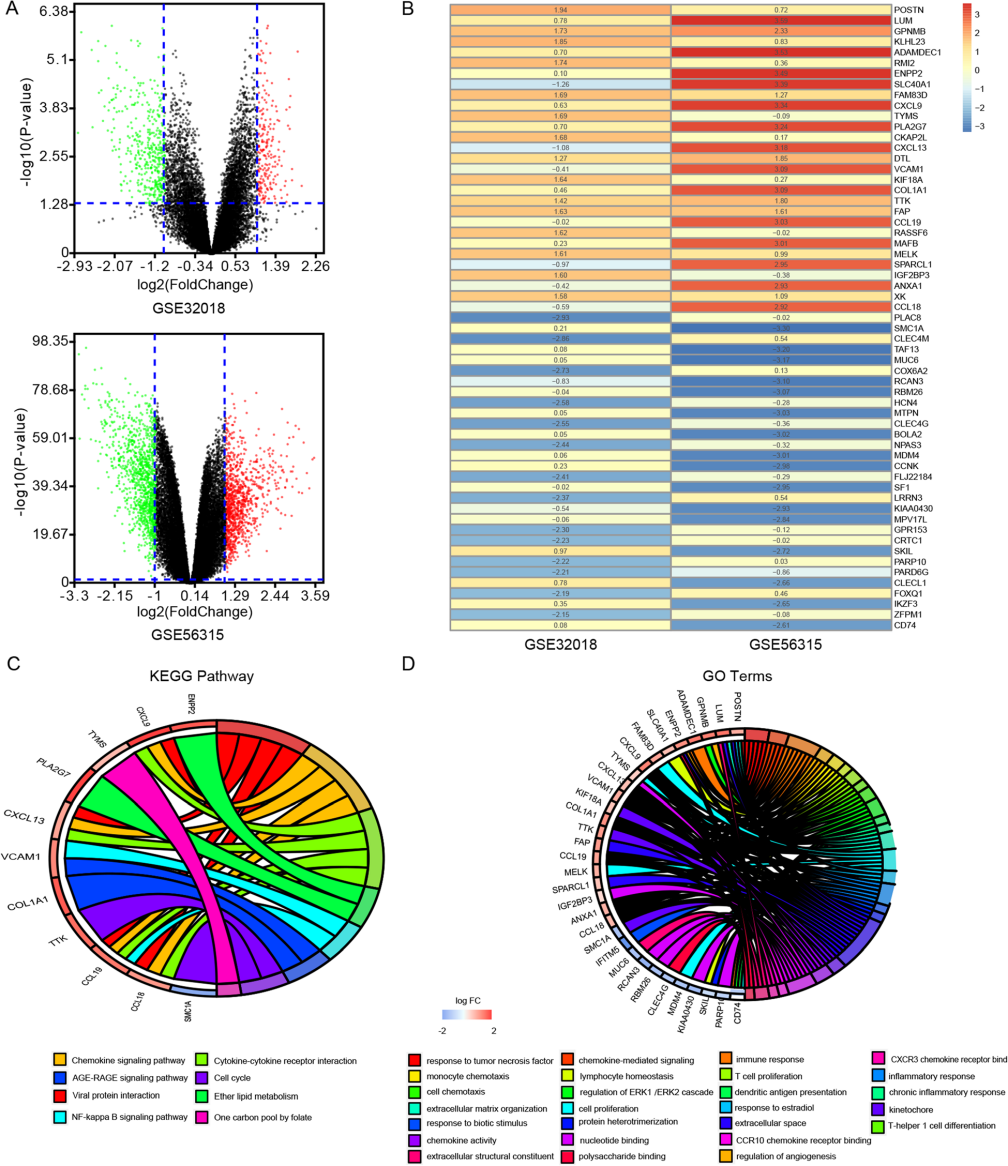
DLBCL-related differentially expressed gene identification. **A**: Volcano plots were used to compare gene expression profiles in the GSE32018 and GSE56315 datasets. Red and green symbols are used to indicate genes that were significantly up- and down-regulated, respectively, as per the following criteria: log2 FC > 1 and adjusted *P*-value < 0.05. **B**: The top 30 upregulated and top 38 downregulated genes in these two datasets were ranked using the Robust Rank Aggreg (RRA) approach. Individual rows corresponding to genes, while columns correspond to datasets. Red and blue represent genes that were up- and down-regulated, respectively, relative to normal tissues. **C**: DEGs were subjected to a KEGG pathway enrichment analysis. **D**: DEGs were subjected to GO term enrichment analyses

The general strategy for the present study is shown in Fig. 1. In total, 208 and 43 up- and down-regulated DLBCL-related DEGs, respectively, were identified in the GSE32018 dataset, while 993 and 1127 up- and down-regulated DEGs, respectively, were identified in the GSE56315 dataset (Fig. 2A). These two datasets were then integrated via the RRA method, revealing 30 and 38 shared DEGs that were up- and down-regulated in DLBCL samples relative to normal tissue samples, respectively (Fig. 2B). KEGG enrichment analysis of these DEGs revealed that they were associated with the cell cycle, the NF-kB signaling pathway, and chemokine signaling (Fig. 2C). GO term enrichment analyses further revealed these DEGs to be associated with immune responses, the regulation of angiogenesis, cell proliferation, chemokine-mediated signaling, and cellular responses to tumor necrosis factor (Fig. 2D).

### Detection of potential diagnostic biomarkers of DLBCL

**Fig. 3:**
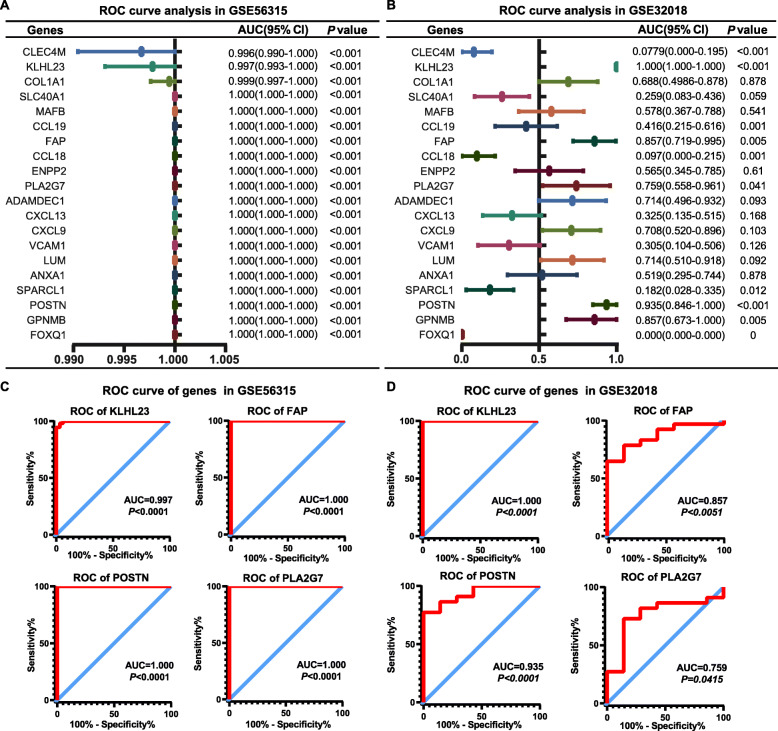
Identification of key DLBCL-related diagnostic biomarkers. **A**: The top 20 genes in the GSE56315 database were subjected to ROC curve analyses. **B**: The top 20 genes in the GSE32018 database were subjected to ROC curve analyses. **C** and **D**: ROC curves were used to assess the specificity and sensitivity of the indicated genes from these two datasets as diagnostic biomarkers of DLBCL. Red represents sensitive curves, blue indicates identify lines. The x-axis represents the false positive rate, and is shown as ‘1-Specifcity’. The y-axis indicates true positive rate, and is shown as ‘Sensitivity’

ROC analyses were subsequently conducted for each of the 68 DEGs identified above, AUC values were used to assess the diagnostic sensitivity and specificity of these genes between DLBCL and normal tissues in the GSE56315 datasets. The top 20 more predictive of these DEGs, which yielded AUC values > 0.996, are shown in Fig. 3A. In order to validate the diagnostic value of these 20 DEGs, these same ROC analyses were repeated using the GSE32018 dataset (Fig. 3B). A subset of these DEGs including KLHL23, FAP, PLA2G7, POSTN, and GPNMB, exhibited high AUC values, suggesting that they may be of significant value as diagnostic biomarkers of DLBCL (Fig. 3C-D).

### WGCNA and identification of key modules

**Fig. 4:**
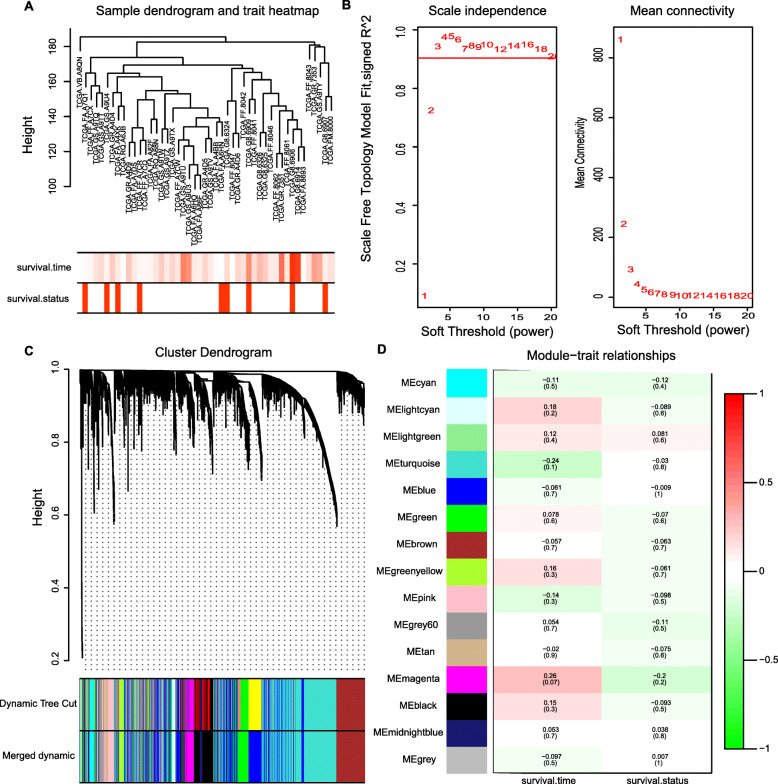
Weighted co-expression network construction and analysis. **A**: A hierarchical clustering dendrogram was used to arrange 48 TCGA database samples, with survival time and survival outcomes being shown at the bottom of this dendrogram. **B**: Scale-free fit index and the mean connectivity analyses. **C**: A clustering dendrogram was employed to identify gene co-expression modules based upon average hierarchical linkages. **D**: A Module-Trait Relationship matrix was generated, with rows and columns corresponding to module eigengenes and clinical traits, respectively, and with correlations and *P*-values being represented in individual boxes

We next employed a systematic WGCNA approach to the unsupervised classification of these data, classifying DEGs into modules based upon their expression patterns [21]. Genes that exhibit highly correlated expression patterns are grouped into the same modules, as they are predicted to have identical or closely related biological functions, allowing for the direct assessment of the functional relevance of individual modules [22]. We conducted this WGCNA analysis based upon gene expression and survival data from 48 DLBCL patients in the TCGA database (Fig. 4A), and a scale-free network was constructed using a power of β = 5 as a soft-threshold (Fig. 4B). This approach ultimately grouped DEGs into 15 different modules that were identified and assigned different colors using a merged dynamic tree cut (Fig. 4C). The gray-colored module contained all genes that were not clustered with one another, and these genes were not analyzed further in this study. Correlations between module eigengene values and sample clinical traits are shown in Fig. 4D. The magenta module was found to be most closely associated with DLBCL patient prognosis, although this association was not significant. (*R*^*2*^ = 0.26, *P* = 0.07 with survival time; *R*^*2*^ = − 0.2, *P* = 0.2 with survival status). Notably, this magenta module included the PLA2G7 gene, which we had identified as a promising diagnostic biomarker in our above analyses. Indeed, PLA2G7 was a hub gene within this magenta module (MM = 0.84 and GS =0.17, genes in magenta module are shown in Supplemental Table S1), and it has previously been shown to function as a key regulator of oncogenesis and inflammation [11]. The expression and functionality of PLA2G7 in DLBCL, however, remains to be fully clarified.

### Elevated PLA2G7 expression is linked to the DLBCL tumor microenvironment

**Fig. 5:**
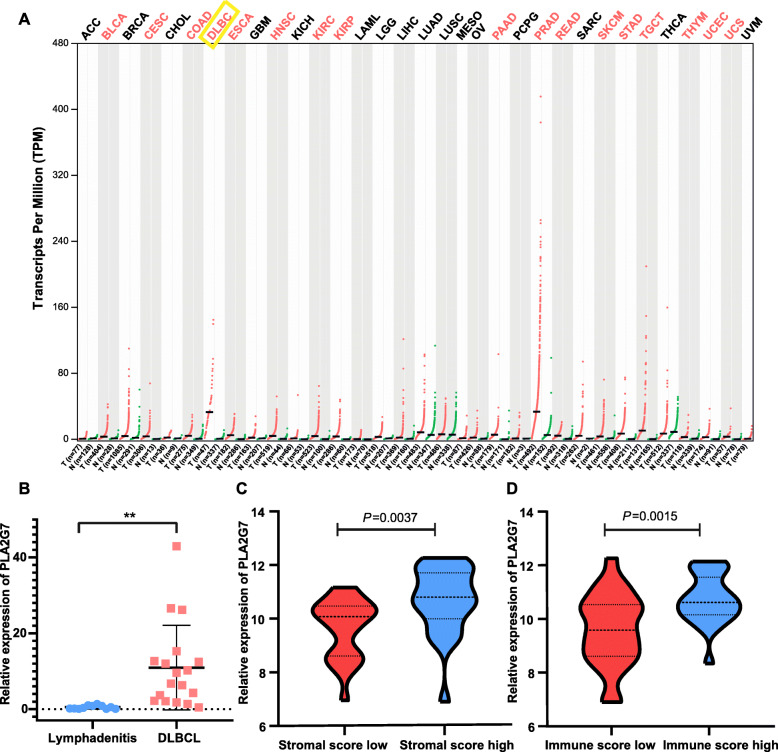
The association between PLA2G7 and tumor progression. **A**: PLA2G7 overexpression was evident in 17 tumor tissue types, including DLBCL, relative to normal tissues as determined through GEPIA analyses. **B**: PLA2G7 expression in DLBCL patient samples and benign lymphadenitis tissue samples was compared. **C** and **D**: PLA2G7 expression in DLBCL samples with different stromal and immune scores was compared based upon the TCGA database

**Fig. 6:**
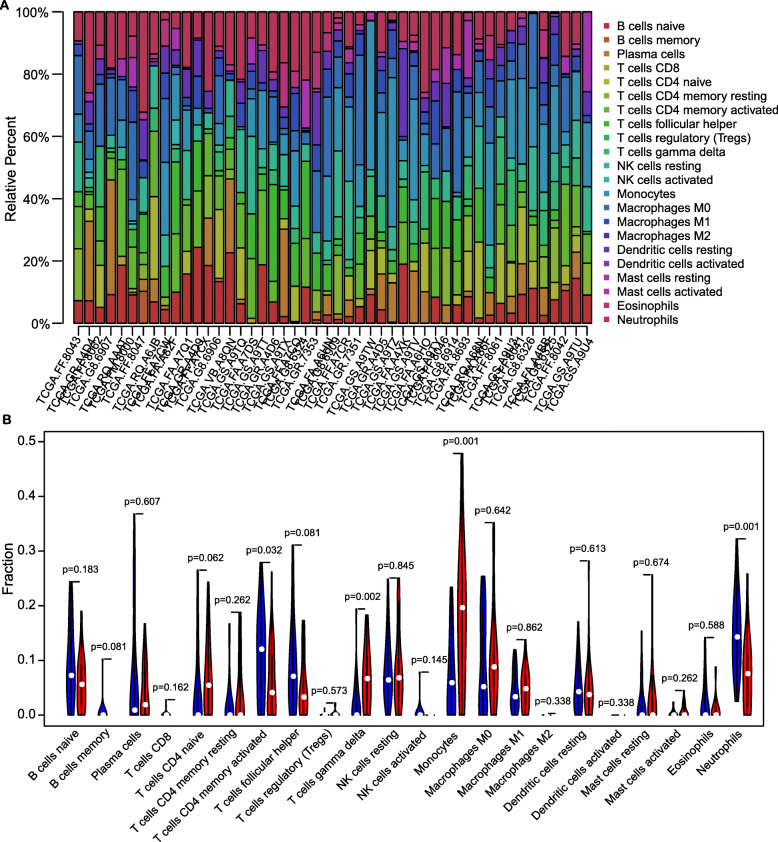
The relationship between PLA2G7 expression and the tumor microenvironment. **A**: Tumor-infiltrating immune cells profiles for 48 patients in the TCGA were established. **B**: Violin plots were used to represent 22 immune cell types in low- and high- PLA2G7 patient cohorts, which are shown in blue and red, respectively

To explore the mechanistic role of PLA2G7 in the context of tumor progression, we next conducted a GEPIA-based conjoint analysis of 33 tumor types within the TCGA and GTEx databases [23]. These analyses revealed that PLA2G7 expression was significantly elevated in 17 tumor types, including DLBCL, relative to corresponding normal tissue controls (Fig. 5A). We then conducted a qRT-PCR-based comparison of PLA2G7 mRNA expression in DLBCL patient tumor tissues and benign lymphadenitis patient tissues, leading us to confirm that this gene is highly expressed in DLBCL tissues (Fig. 5B, mean = 14.95, 95% confidence interval = 4.661–25.23). Clinicopathological features in DLBCL patients were shown in Supplemental Table S2. Further analyses of the TCGA database revealed that high PLA2G7 expression (greater than the median value (cut-off =10.32)) was associated with higher stromal (*P* = 0.021) and immune (*P* = 0.004) scores (Fig. 5C-D). In contrast, these expression levels were not correlated with tumor size or stage (Table 1). These findings suggest that PLA2G7 expression is associated with intratumoral heterogeneity and the composition of the tumor microenvironment (TME). The TME consists of stromal cells, tumor cells, immune cells, and a milieu of cytokines and other compounds that play critical roles in modulating cancer onset and progression [24]. To fully explore the association between PLA2G7 expression and immune cell infiltration in the TME, CIBERSORT [25], which can be used to analyze the proportions of 22 of tumor-infiltrating immune cell (TIIC) types based upon RNA-seq data, was used to compare TIIC profiles in PLA2G7-low and -high DLBCL patients from the TCGA cohort (Fig. 6A). This analysis revealed that PLA2G7-high patients exhibited significantly higher proportions of monocytes and gamma delta T cells and lower frequencies of neutrophils relative to PLA2G7-low patients (Fig. 6B).

**Table 1:** Correlations between PLA2G7 and clinicopathological characteristics of DLBCL based on TCGA database

Characteristics	PLA2G7 expression		*P-value*
	High,no.cases	Low,no.cases	
Age (years)			
≤ 57	13	11	
> 57	11	13	0.564
Gender			
Female	11	15	
Male	13	9	0.247
Stromal score			
Low	8	16	
High	16	8	0.021
Immune score			
Low	7	17	
High	17	7	0.004
Maximum tumor dimension			
NA	6	4	
≤ 3	8	12	
> 3	10	8	0.491
Primary therapy outcome			
NA	1	4	
CR	18	17	
PR	2	0	
SD	1	1	
PD	2	2	0.43
Tumor stage			
NA	2	4	
I	4	4	
II	9	8	
III	4	1	
IV	5	7	0.552

### PLA2G7 promotes DLBCL cell proliferation and migration and inhibits apoptosis in vitro

**Fig. 7:**
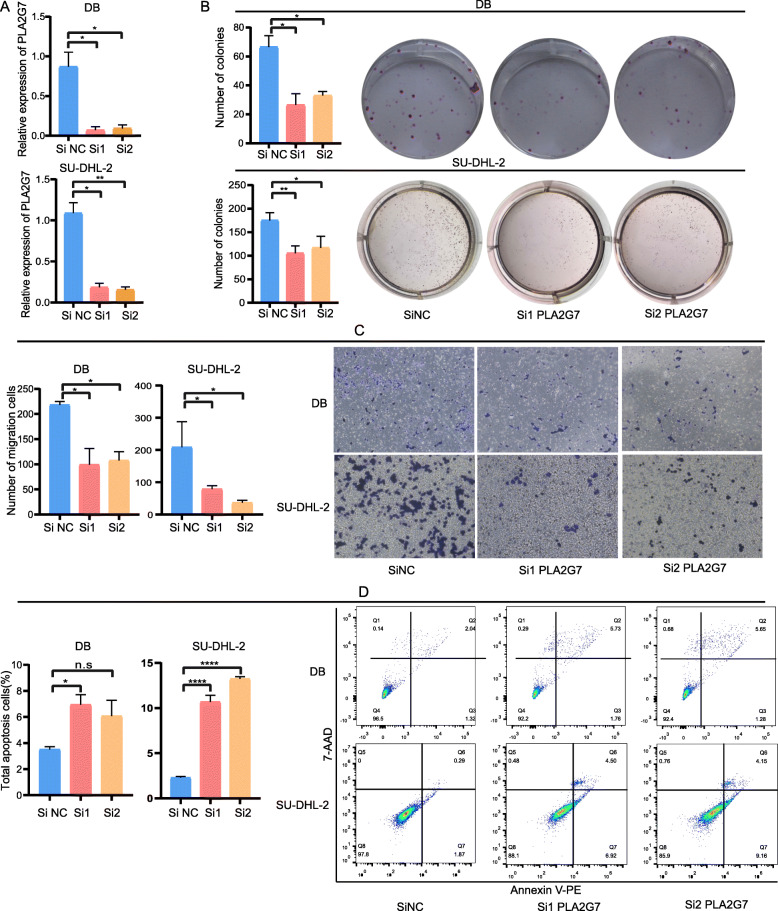
PLA2G7 drives the in vitro migration and proliferation of DLBCL cells and inhibits their apoptotic death. **A**: PLA2G7 mRNA expression in DB and SU-DHL-2 cells following PLA2G7 siRNA transfection was assessed. **B**: Negative Control (NC) siRNA-, and PLA2G7 siRNA- transfected DB and SU-DHL-2 cells were evaluated in a colony formation assay. **C**: Cells prepared as in (**B**) were evaluated in a migration assay. **D:** Cells prepared as in (**B**) were assessed in an apoptosis assay. n.s, not significant, *P ≤ 0·05, **P < 0.01, ***P < 0.001, ****P < 0.0001

To directly interrogate the role of PLA2G7 in DLBCL cells, we knocked down this gene in DB and SU-DHL-2 cell line and confirmed successful knockdown via qRT-PCR using two different siRNA constructs (Fig. 7A). Knockdown of PLA2G7 suppressed the ability of these DLBCL tumor cells to migrate and form colonies (Fig. 7B and C). Notably, such knockdown also markedly enhanced the death of these two DLBCL cells as measured in an Annexin V/7AA-D staining assay (Fig. 7D). Together, these data provide clear evidence in support of a model wherein PLA2G7 plays an oncogenic role in DLBCL by inhibiting tumor cell apoptotic death while simultaneously enhancing proliferation and migration.

### Effect of darapladib on DLBCL proliferation and apoptosis

**Fig. 8:**
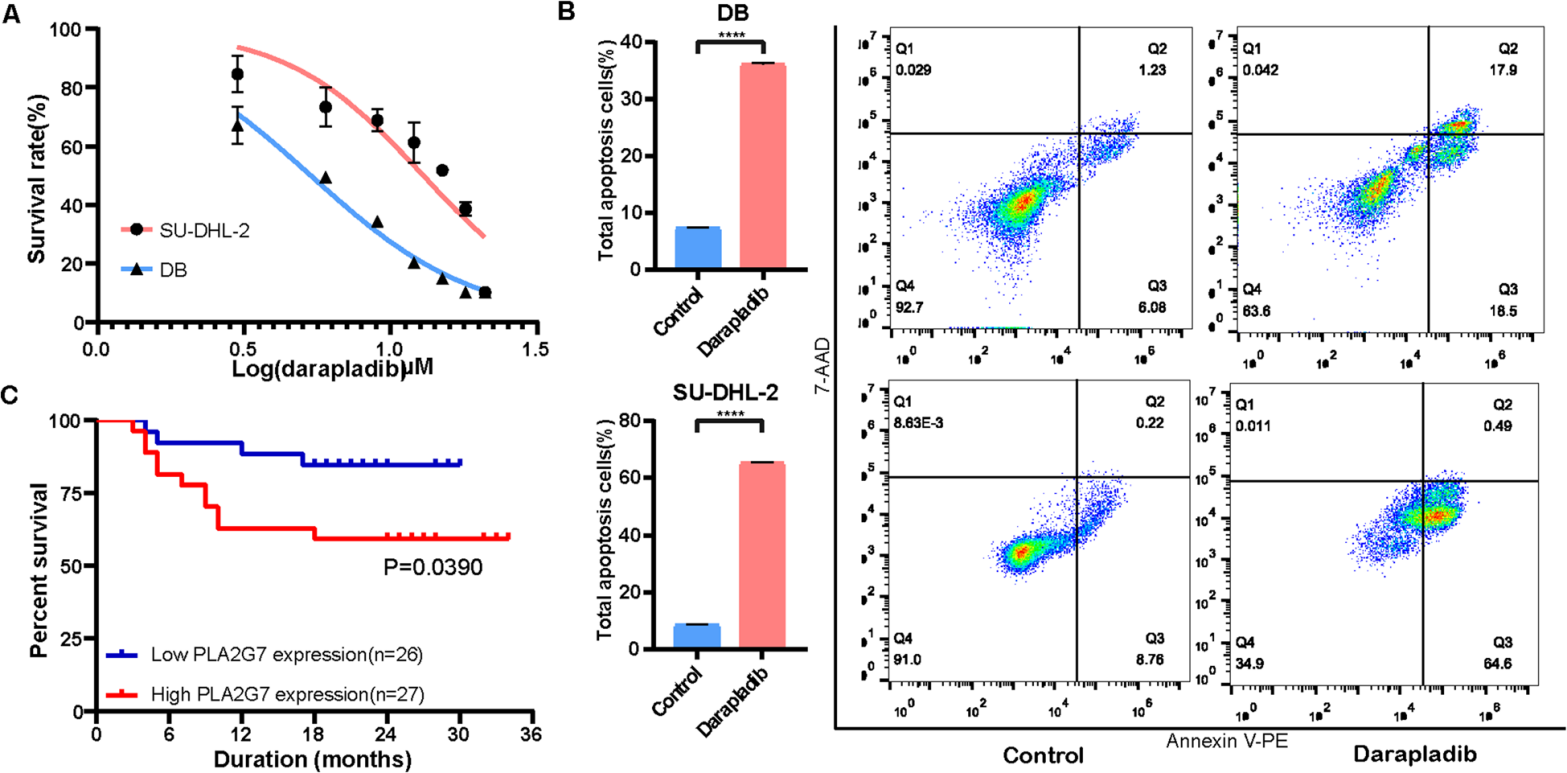
Darapladib inhibits DLBCL cells proliferation and induced apoptosis. **A**: DB and SU-DHL-2 cells were incubated with increasing concentrations of darapladib for 72 h. Cell viability was determined with the CCK8 assay. **B**: Darapladib induced apoptosis in DB and SU-DHL-2 cells. **C**: The expression of PLA2G7 was significantly related to overall survival of DLBCL patients. Red and blue lines indicate high-expression and low-expression groups, respectively. ****P < 0.0001

To further explore the role of PLA2G7 as a therapeutic target, the cytotoxicity effect of darapladib, a specific inhibitor of PLA2G7, on DLBCL cells was investigated. DLBCL cells were treated with various concentrations of darapladib for 72 h, and the cell proliferation ability was detected with a CCK8 assay. The results suggest that darapladib significantly attenuated the DB and SU-DHL-2 cells viability, with IC50 values at 5.33 μM and 12.92 μM, respectively (Fig. 8A). In addition, cell apoptosis analysis indicates darapladib resulted in a remarkable increase in the number of apoptotic cells when DB and SU-DHL-2 cells were treated with 5.33 μM and 12.92 μM darapladib for 48 h, respectively (Fig. 8B). Consequently, darapladib could noticeably restrained the DLBCL cell viability and induced apoptosis.

### Clinical and prognostic significance of PLA2G7

To further explore the clinical significance of PLA2G7, the relationship between PLA2G7 mRNA expression in peripheral blood and clinical characteristics of 53 DLBCL patients were analyzed (Table 2). The median PLA2G7 mRNA levels in DLBCL samples were regarded as the cut-off. High expression of PLA2G7 correlated with high levels of serum B2m (*P* = 0.0039), and low levels of serum albumin (*P* = 0.005) and CD10 positive (*P* = 0.006). In contrast, low expression of PLA2G7 was found to be associated with the non-GCB subtype (*P* = 0.034). Furthermore, survival analysis results indicate that DLBCL patients with high expression of PLA2G7 are associated with a worse prognosis (Fig. 8C).

**Table 2:** Correlations between PLA2G7 and clinicopathological characteristics of DLBCL peripheral blood. (GCB: germinal centre B-cell like; IPI: international prognostic index; CR: complete response; B2m: Recombinant Human beta-2-Microglobulin; LDH: lactate dehydrogenase; BCL: B-cell lymphoma)

Characteristics	PLA2G7 expression		*P-value*
	High,no.cases(≥1.46)	Low,no.cases(< 1.46)	
Age (years)			0.477
≤ 54	14	16	
> 54	13	10	
Gender			0.206
Female	14	9	
Male	13	17	
stage			0.107
I–II	5	10	
III–IV	22	16	
Subtype			0.034
GCB	13	7	
Non-GCB	14	19	
IPI score			0.467
0–2	21	23	
> 3	6	3	
CR after first-line chemotheray			0.318
yes	14	17	
no	13	9	
A/B symptoms			0.810
A	20	20	
B	7	6	
Low serum albumin			0.005
yes	11	2	
no	16	24	
High serum B2m			0.039
yes	19	11	
no	8	15	
High serum LDH			0.335
yes	15	11	
no	12	15	
CD10			0.006
positive	15	5	
negative	12	21	
CD20			0.978
positive	26	25	
negative	1	1	
BCL-2			0.420
positive	25	22	
negative	2	4	
BCL-6			0.175
positive	24	19	
negative	3	7	

## Discussion

DLBCL is a common and highly heterogeneous form of NHL associated with high morbidity and mortality rates [26]. While roughly half of DLBCL cases can be cured via standard chemotherapy, treated relapsed or refractory DLBCL remains challenging [27]. It is thus essential that prognostic and diagnostic biomarkers of DLBCL be identified in order to guide patient detection and treatment. Herein, we therefore sought to identify potential prognostic and diagnostic biomarkers of DLBCL. In total, we identified 30 and 38 genes that were significantly up- and down-regulated, respectively, in DLBCL samples from two GEO datasets. Functional enrichment analyses suggested that these DEGs were associated with angiogenesis, cell proliferation, the immune response, and other key tumor progression-related pathways. Of these 68 DEGs, five were identified as promising diagnostic biomarkers of DLBCL through ROC curve analyses.

We next utilized the TCGA database in order to conduct WGCNA analyses exploring the relationship between gene expression profiles and DLBCL patient prognosis. Through this approach, we identified a key gene module that was related to patient outcomes. PLA2G7 was a hub gene within this module, and also exhibited favorable diagnostic utility in the above ROC curve analyses. Prior research indicates that PLA2G7 is a tumor-associated macrophage-derived factor that plays a key role in the regulation of tumor cell migration. In nasopharyngeal carcinoma (NPC) cells, there is evidence that PLA2G7 can enhance tumor cell migration and survival [28], and similar findings have also been observed in PCa cells [11]. Further research has led to the identification of this gene as a biomarker of primary and metastatic PCa, and a viable therapeutic target in patients with ERG positive PCa [29]. In the GTex and TCGA databases, PLA2G7 was found to be upregulated in 17 different tumor types. Consistent with prior studies of PCa and NPC, we also determined that PLA2G7 promoted DLBCL cell proliferation and migration while suppressing the apoptotic death of these cells.

The tumor microenvironment includes macrophages, dendritic cells, T helper cells, T cytotoxic cells, and reactive B lymphocytes. Shain et al. [30] previously demonstrated that B cell tumor interactions with the local TME can influence tumor cell behavior by controlling the oncogenesis’s growth and progression. Among these components, tumor-associated macrophages (TAM) were found to play a major role. The data generated in the present study further suggest that PLA2G7 expression may be associated with DLBCL tumor stromal and immune scores. Kua et al. [31, 32] reported that the CIBERSORT algorithm was used to analyze the DLBCL immune cell infiltration in the TME. As such, we explored the association between PLA2G7 expression and TIICs, revealing that patients expressing high levels of this gene also exhibited increased monocyte infiltration. Monocytes develop into macrophages in tumors, and are associated with poor prognosis in DLBCL patients due to IL-34 production [33]. It is reported that the OS and PFS of DLBCL patients with high expression of TAMs are often poor, and there is a positive correlation with the peripheral absolute monocyte count (AMC) [34]. Therefore, AMC is a useful prognostic marker that reflects the status of the tumor microenvironment (TME) in DLBCL. Increased tumor-infiltrating monocyte numbers may thus be responsible for the relationship between high PLA2G7 expression and poor DLBCL patient outcomes. Together, these data, therefore, offer insight into potential immunotherapeutic treatment strategies for this cancer type.

Notably, our study focused on the identification of PLA2G7 as a novel biomarker for DLBCL. However, it had some limitations. First, the sample size obtained from TCGA to validate the GEO data sets was not large, the data in TCGA about DLBCL lacked normal samples as controls and complete survival information was lacking in some cases. Future studies are required and should include a large sample size to validate such observations. Also, our study focused on bioinformatics methods and in vitro experiments to screen candidate genes for DLBCL and identified PLA2G7 as a novel biomarker, the functional role of PLA2G7 could be explored further to determine tumor cell migration using in vivo studies and a detailed mechanistic approach. Future studies are required and should include these approaches.

In summary, we herein identified PLA2G7 as a biomarker that is upregulated in DLBCL and that is related to the enhancement of DLBCL cell proliferation, invasion, and tumor microenvironmental composition. These findings suggest that PLA2G7 may not only be a diagnostic and prognostic biomarker of DLBCL, but also a viable therapeutic target for the improvement of patient outcomes.

## Supplementary Information

**Additional file 1.**

**Additional file 2.**

## Data Availability

The datasets used or analysed during the current study are available from the corresponding author on reasonable request.

## Abbreviations

DLBCL: Diffuse large B-cell lymphoma
GEO: Gene expression omnibus
WCGNA: Weighted gene co-expression network
TCGA: The cancer genome atlas
DEG: Differentially expressed gene
GO: Gene ontology
KEGG: Kyoto encyclopedia of genes and genomes
ROC: Receiver operating characteristic
RRA: RobustRankAggreg
