# Supplementary material for: Identification of PLA2G7 as a novel biomarker of diffuse large B cell lymphoma
Source: BMC Cancer. 2021 Aug 17;21:927. doi: 10.1186/s12885-021-08660-4 (PMC8369790; doi:10.1186/s12885-021-08660-4)
Supplement: Supplementary file 1 — Additional file 1. [file 12885_2021_8660_MOESM1_ESM.docx]

Supplemental Table S1

Genes in magenta group

| geneSymbol | moduleColor | GS.Surtime | p.GS.Surtime | MM.magenta | p.MM.magenta |
| --- | --- | --- | --- | --- | --- |
| FAIM2 | magenta | 0.419129 | 0.003364 | 0.552977 | 5.56E-05 |
| SIGLEC12 | magenta | -0.40415 | 0.004842 | -0.41056 | 0.004152 |
| AMZ1 | magenta | 0.396497 | 0.005795 | 0.674126 | 2.05E-07 |
| DQX1 | magenta | -0.37061 | 0.010335 | -0.41998 | 0.003294 |
| ANKRD2 | magenta | 0.363479 | 0.012028 | 0.821961 | 1.42E-12 |
| TREM2 | magenta | 0.362667 | 0.012234 | 0.76861 | 2.82E-10 |
| ENTHD1 | magenta | 0.354944 | 0.014359 | 0.68881 | 8.68E-08 |
| CEBPA | magenta | 0.351378 | 0.015442 | 0.831069 | 4.83E-13 |
| CSF2RA | magenta | 0.351104 | 0.015528 | 0.866911 | 3.35E-15 |
| FAM18A | magenta | 0.350961 | 0.015573 | 0.356389 | 0.01394 |
| OSGIN1 | magenta | 0.343501 | 0.018082 | 0.850785 | 3.68E-14 |
| SPHK1 | magenta | 0.337117 | 0.020492 | 0.753748 | 9.64E-10 |
| CAPG | magenta | 0.331186 | 0.022969 | 0.881566 | 2.85E-16 |
| CPNE9 | magenta | 0.32966 | 0.023645 | 0.57112 | 2.76E-05 |
| NUPR1 | magenta | 0.322944 | 0.026824 | 0.839236 | 1.73E-13 |
| CDA | magenta | 0.319886 | 0.028385 | 0.85614 | 1.72E-14 |
| APOE | magenta | 0.317677 | 0.029559 | 0.853078 | 2.66E-14 |
| SDSL | magenta | 0.316743 | 0.030067 | 0.74645 | 1.71E-09 |
| BIRC7 | magenta | 0.316572 | 0.030161 | 0.668973 | 2.75E-07 |
| IL3RA | magenta | 0.312684 | 0.032362 | 0.720028 | 1.17E-08 |
| CCL15 | magenta | 0.3084 | 0.034939 | 0.609681 | 5.39E-06 |
| MYO18B | magenta | -0.30675 | 0.035974 | -0.29778 | 0.042067 |
| FAM20C | magenta | 0.304245 | 0.037598 | 0.690713 | 7.74E-08 |
| TNFRSF4 | magenta | 0.297859 | 0.042008 | 0.549653 | 6.29E-05 |
| LCN8 | magenta | -0.29413 | 0.044774 | -0.38926 | 0.006844 |
| PRSS36 | magenta | 0.292913 | 0.045706 | 0.826981 | 7.90E-13 |
| HES2 | magenta | 0.292638 | 0.045919 | 0.809471 | 5.68E-12 |
| APOC1 | magenta | 0.29169 | 0.04666 | 0.772866 | 1.95E-10 |
| CHIT1 | magenta | 0.290729 | 0.047421 | 0.823399 | 1.20E-12 |
| GADD45G | magenta | 0.287229 | 0.050279 | 0.7011 | 4.06E-08 |
| PTGDS | magenta | 0.286498 | 0.050893 | 0.709531 | 2.36E-08 |
| ATP1A4 | magenta | 0.281036 | 0.055675 | 0.781332 | 9.15E-11 |
| AK3L1 | magenta | 0.272832 | 0.063532 | 0.405499 | 0.004688 |
| SDC4 | magenta | 0.270429 | 0.065994 | 0.835811 | 2.68E-13 |
| PLEKHG3 | magenta | 0.268768 | 0.067739 | 0.852109 | 3.06E-14 |
| CYP27A1 | magenta | 0.267866 | 0.068702 | 0.876016 | 7.52E-16 |
| LGALS2 | magenta | 0.264605 | 0.072275 | 0.606932 | 6.10E-06 |
| LOC283663 | magenta | -0.26419 | 0.072745 | -0.34249 | 0.018446 |
| FBP1 | magenta | 0.264017 | 0.072935 | 0.55086 | 6.01E-05 |
| KCNF1 | magenta | 0.261514 | 0.075797 | 0.478943 | 0.00066 |
| VMO1 | magenta | 0.258578 | 0.079264 | 0.643843 | 1.05E-06 |
| CTSK | magenta | 0.25532 | 0.083257 | 0.806506 | 7.77E-12 |
| PLA2G2D | magenta | 0.252512 | 0.086825 | 0.667979 | 2.90E-07 |
| POF1B | magenta | 0.250931 | 0.088884 | 0.305982 | 0.036467 |
| TMEM119 | magenta | 0.249268 | 0.091092 | 0.678537 | 1.59E-07 |
| C8G | magenta | 0.249157 | 0.091241 | 0.590818 | 1.23E-05 |
| ITIH3 | magenta | 0.24793 | 0.092899 | 0.58 | 1.93E-05 |
| MMP9 | magenta | 0.247364 | 0.093671 | 0.770657 | 2.37E-10 |
| C9orf98 | magenta | 0.245855 | 0.095756 | 0.695807 | 5.66E-08 |
| UNC13A | magenta | 0.245387 | 0.09641 | 0.69455 | 6.12E-08 |
| RTN1 | magenta | 0.243817 | 0.098628 | 0.691801 | 7.24E-08 |
| TM4SF19 | magenta | 0.241768 | 0.101581 | 0.683336 | 1.20E-07 |
| NDP | magenta | 0.240956 | 0.10277 | 0.728721 | 6.36E-09 |
| SIGLEC15 | magenta | 0.240391 | 0.103604 | 0.74099 | 2.59E-09 |
| TM7SF4 | magenta | 0.237979 | 0.107221 | 0.783906 | 7.22E-11 |
| C9orf45 | magenta | -0.23692 | 0.108845 | -0.58617 | 1.50E-05 |
| TIFAB | magenta | 0.23673 | 0.109132 | 0.672381 | 2.27E-07 |
| CES1 | magenta | 0.23671 | 0.109164 | 0.804251 | 9.83E-12 |
| LYZ | magenta | 0.235164 | 0.111565 | 0.857859 | 1.33E-14 |
| CYP4F22 | magenta | 0.234921 | 0.111947 | 0.534487 | 0.000109 |
| EMID2 | magenta | 0.234697 | 0.112299 | 0.716799 | 1.45E-08 |
| DDX11L2 | magenta | 0.232031 | 0.116556 | 0.30115 | 0.039685 |
| RGS6 | magenta | -0.23074 | 0.118657 | -0.2535 | 0.085561 |
| ACP5 | magenta | 0.228736 | 0.121985 | 0.735022 | 4.03E-09 |
| CBS | magenta | 0.227813 | 0.12354 | 0.430984 | 0.002492 |
| PLAUR | magenta | 0.227617 | 0.123873 | 0.799793 | 1.55E-11 |
| CALML5 | magenta | 0.226905 | 0.125084 | 0.335309 | 0.021222 |
| OSCAR | magenta | 0.226212 | 0.126272 | 0.738907 | 3.03E-09 |
| ITGB1BP3 | magenta | 0.224064 | 0.130009 | 0.394062 | 0.006131 |
| PIR | magenta | 0.223596 | 0.130834 | 0.781156 | 9.30E-11 |
| CCDC103 | magenta | 0.221869 | 0.133913 | 0.662617 | 3.90E-07 |
| SLC47A1 | magenta | 0.219927 | 0.137439 | 0.595845 | 9.93E-06 |
| NCCRP1 | magenta | 0.218267 | 0.140507 | 0.252347 | 0.087037 |
| OR13A1 | magenta | -0.21728 | 0.142357 | -0.30406 | 0.037719 |
| MMP14 | magenta | 0.217208 | 0.142491 | 0.811416 | 4.61E-12 |
| LOC283050 | magenta | 0.215653 | 0.14544 | 0.780553 | 9.82E-11 |
| ALOX15B | magenta | 0.215137 | 0.14643 | 0.85947 | 1.05E-14 |
| MMP25 | magenta | 0.214717 | 0.14724 | 0.664234 | 3.57E-07 |
| MGAT3 | magenta | -0.21088 | 0.154782 | -0.39558 | 0.00592 |
| MAGEB1 | magenta | -0.20984 | 0.15687 | -0.22133 | 0.134884 |
| CTNND2 | magenta | 0.209453 | 0.157656 | 0.692828 | 6.80E-08 |
| PLIN4 | magenta | -0.20935 | 0.157868 | -0.29745 | 0.042303 |
| LMO7 | magenta | -0.20721 | 0.162249 | -0.52009 | 0.000179 |
| IL22RA1 | magenta | -0.20696 | 0.162772 | -0.24469 | 0.097385 |
| SPATA12 | magenta | 0.206234 | 0.164285 | 0.730044 | 5.78E-09 |
| CPM | magenta | 0.202587 | 0.172039 | 0.776177 | 1.46E-10 |
| C4orf34 | magenta | -0.20243 | 0.172379 | -0.22313 | 0.131662 |
| TRIM72 | magenta | -0.20141 | 0.174605 | -0.40355 | 0.004911 |
| RTN4R | magenta | 0.201229 | 0.174995 | 0.551211 | 5.94E-05 |
| GPNMB | magenta | 0.200948 | 0.175609 | 0.802585 | 1.17E-11 |
| FABP3 | magenta | 0.200551 | 0.176482 | 0.523962 | 0.000157 |
| DNAJB13 | magenta | -0.19858 | 0.180867 | -0.22782 | 0.123529 |
| C15orf48 | magenta | 0.198196 | 0.181724 | 0.809183 | 5.85E-12 |
| TMEM163 | magenta | 0.198046 | 0.182061 | 0.617724 | 3.73E-06 |
| ZMYND15 | magenta | 0.197578 | 0.183118 | 0.742605 | 2.29E-09 |
| AMICA1 | magenta | 0.194455 | 0.190277 | 0.539376 | 9.15E-05 |
| PKP1 | magenta | 0.190688 | 0.199174 | 0.503798 | 0.000306 |
| CHST6 | magenta | 0.190115 | 0.200552 | 0.572536 | 2.61E-05 |
| LTA | magenta | 0.189671 | 0.201627 | 0.489758 | 0.000476 |
| STEAP3 | magenta | 0.189592 | 0.201817 | 0.778176 | 1.22E-10 |
| UBD | magenta | 0.18835 | 0.204843 | 0.77262 | 1.99E-10 |
| CCRL1 | magenta | -0.18663 | 0.209084 | -0.35689 | 0.013797 |
| ACHE | magenta | 0.186537 | 0.209314 | 0.717521 | 1.38E-08 |
| IGDCC3 | magenta | 0.186392 | 0.209675 | 0.329071 | 0.023911 |
| BEAN | magenta | 0.1856 | 0.211654 | 0.723243 | 9.35E-09 |
| KRT36 | magenta | 0.18458 | 0.214218 | 0.674181 | 2.05E-07 |
| DEPDC6 | magenta | 0.183124 | 0.217921 | 0.270534 | 0.065885 |
| ITIH1 | magenta | 0.182844 | 0.218635 | 0.787912 | 4.96E-11 |
| CLEC4D | magenta | 0.180986 | 0.223434 | 0.679986 | 1.47E-07 |
| LGI2 | magenta | 0.177564 | 0.232457 | 0.570823 | 2.79E-05 |
| UNC13B | magenta | 0.172918 | 0.245104 | 0.600874 | 7.98E-06 |
| ELOVL3 | magenta | -0.17179 | 0.24824 | 0.482521 | 0.000593 |
| VNN3 | magenta | -0.17152 | 0.249001 | 0.272081 | 0.064294 |
| MMP10 | magenta | -0.17062 | 0.251522 | -0.22123 | 0.13507 |
| GDF15 | magenta | 0.170381 | 0.252202 | 0.581064 | 1.85E-05 |
| RALGPS2 | magenta | -0.17031 | 0.252417 | -0.60628 | 6.28E-06 |
| SLMO1 | magenta | -0.16913 | 0.255749 | -0.3116 | 0.033001 |
| APOC1P1 | magenta | 0.167949 | 0.259136 | 0.50837 | 0.000264 |
| PLA2G7 | magenta | 0.167145 | 0.261458 | 0.845653 | 7.44E-14 |
| LOC389634 | magenta | 0.165866 | 0.265177 | 0.80177 | 1.27E-11 |
| RICH2 | magenta | -0.16487 | 0.268093 | -0.34799 | 0.016534 |
| CRYBB1 | magenta | 0.164358 | 0.269607 | -0.24247 | 0.100569 |
| MGC87042 | magenta | -0.16347 | 0.272225 | -0.19919 | 0.179489 |
| BEGAIN | magenta | 0.163371 | 0.272532 | 0.248585 | 0.092011 |
| CYP27B1 | magenta | 0.162314 | 0.27569 | 0.741751 | 2.45E-09 |
| PLAU | magenta | 0.161909 | 0.276907 | 0.721828 | 1.03E-08 |
| PTGES | magenta | 0.161269 | 0.278834 | 0.758224 | 6.72E-10 |
| ORM1 | magenta | 0.160704 | 0.280543 | 0.784259 | 6.98E-11 |
| GPC3 | magenta | 0.160029 | 0.282596 | -0.30008 | 0.040426 |
| SIGLEC14 | magenta | 0.157781 | 0.289502 | 0.360527 | 0.012794 |
| CSTA | magenta | 0.156937 | 0.292122 | 0.681953 | 1.31E-07 |
| LCNL1 | magenta | 0.156738 | 0.292742 | 0.665089 | 3.41E-07 |
| ALDH2 | magenta | 0.155979 | 0.295114 | 0.623827 | 2.80E-06 |
| PGBD5 | magenta | 0.155452 | 0.296769 | 0.515615 | 0.000208 |
| SOD2 | magenta | 0.154128 | 0.300952 | 0.741782 | 2.44E-09 |
| PPP1R1A | magenta | 0.153084 | 0.30428 | 0.65109 | 7.22E-07 |
| BHLHE41 | magenta | 0.15305 | 0.304389 | 0.571223 | 2.75E-05 |
| ZNF215 | magenta | 0.152869 | 0.304967 | -0.37998 | 0.008426 |
| IL4I1 | magenta | 0.152863 | 0.304986 | 0.531259 | 0.000122 |
| DAPL1 | magenta | 0.152567 | 0.305934 | 0.482838 | 0.000587 |
| CLEC4E | magenta | 0.151643 | 0.308907 | 0.831604 | 4.52E-13 |
| CAMK1G | magenta | 0.149624 | 0.315468 | 0.775744 | 1.51E-10 |
| C5AR1 | magenta | 0.148504 | 0.319145 | 0.747466 | 1.58E-09 |
| RUNDC3B | magenta | -0.1473 | 0.32314 | -0.38695 | 0.007212 |
| MCHR1 | magenta | 0.146369 | 0.326229 | 0.633215 | 1.78E-06 |
| SLC6A7 | magenta | 0.145614 | 0.32876 | 0.807571 | 6.94E-12 |
| C6orf164 | magenta | -0.14503 | 0.330707 | -0.26599 | 0.070745 |
| POPDC3 | magenta | 0.14401 | 0.334171 | 0.673573 | 2.12E-07 |
| PTGIR | magenta | 0.142549 | 0.339152 | 0.544956 | 7.48E-05 |
| CHI3L1 | magenta | 0.14221 | 0.340314 | 0.739818 | 2.83E-09 |
| CLEC6A | magenta | 0.139056 | 0.35124 | 0.671985 | 2.32E-07 |
| PAX8 | magenta | 0.13666 | 0.359682 | 0.470827 | 0.000838 |
| KCNJ5 | magenta | 0.136358 | 0.360755 | 0.455362 | 0.001299 |
| CRABP1 | magenta | 0.135995 | 0.362046 | 0.583095 | 1.70E-05 |
| SCARA5 | magenta | 0.135623 | 0.363371 | 0.704067 | 3.36E-08 |
| HSPA1B | magenta | 0.134252 | 0.36829 | 0.418422 | 0.003423 |
| LAD1 | magenta | 0.134241 | 0.368327 | 0.70599 | 2.97E-08 |
| HTRA4 | magenta | 0.133271 | 0.371831 | 0.763237 | 4.45E-10 |
| DNASE2B | magenta | 0.130758 | 0.380996 | 0.637898 | 1.41E-06 |
| MEP1A | magenta | 0.1306 | 0.381577 | 0.576529 | 2.22E-05 |
| ORM2 | magenta | 0.129243 | 0.386584 | 0.775923 | 1.49E-10 |
| OSTalpha | magenta | 0.127529 | 0.392968 | 0.637773 | 1.42E-06 |
| CCL3L1 | magenta | 0.124741 | 0.403481 | 0.515942 | 0.000206 |
| RARRES1 | magenta | 0.123888 | 0.406726 | 0.470169 | 0.000854 |
| FCN1 | magenta | 0.123287 | 0.409024 | 0.440152 | 0.001961 |
| EGF | magenta | -0.12172 | 0.415049 | -0.35717 | 0.013717 |
| FAM19A4 | magenta | 0.1189 | 0.426023 | 0.463208 | 0.001043 |
| RTDR1 | magenta | -0.11715 | 0.432908 | -0.55343 | 5.46E-05 |
| CLLU1OS | magenta | -0.11368 | 0.446766 | 0.238704 | 0.106125 |
| VNN1 | magenta | 0.113396 | 0.447899 | 0.725617 | 7.92E-09 |
| G0S2 | magenta | 0.113226 | 0.448585 | 0.675015 | 1.95E-07 |
| SDR42E1 | magenta | 0.113125 | 0.448992 | -0.29947 | 0.040859 |
| LIN7A | magenta | 0.112199 | 0.452737 | 0.550183 | 6.17E-05 |
| DNAJC5B | magenta | 0.10919 | 0.465023 | 0.532583 | 0.000116 |
| CLDN7 | magenta | 0.108816 | 0.466564 | 0.481078 | 0.000619 |
| B4GALT6 | magenta | 0.108266 | 0.468834 | -0.40641 | 0.004588 |
| ATP6V0D2 | magenta | 0.107663 | 0.471325 | 0.675143 | 1.94E-07 |
| IRX6 | magenta | 0.107395 | 0.472439 | 0.682307 | 1.28E-07 |
| ABCC3 | magenta | 0.106454 | 0.476347 | 0.721482 | 1.06E-08 |
| SLC7A11 | magenta | 0.104555 | 0.484295 | 0.666782 | 3.10E-07 |
| LAMB3 | magenta | 0.103112 | 0.49038 | 0.711052 | 2.13E-08 |
| CAMK2A | magenta | 0.101279 | 0.498163 | 0.628817 | 2.21E-06 |
| ITGAM | magenta | 0.101252 | 0.49828 | 0.649027 | 8.04E-07 |
| ATP2B2 | magenta | 0.098282 | 0.51103 | 0.395561 | 0.005923 |
| MAOA | magenta | 0.097882 | 0.512756 | 0.472916 | 0.000788 |
| FAM49A | magenta | 0.097642 | 0.513798 | 0.399887 | 0.005354 |
| TPD52 | magenta | 0.096826 | 0.517339 | -0.43712 | 0.002125 |
| PTGS1 | magenta | 0.096345 | 0.51943 | 0.439925 | 0.001973 |
| TTC39A | magenta | -0.09595 | 0.521148 | 0.531113 | 0.000123 |
| FAM129C | magenta | -0.09431 | 0.528324 | -0.40921 | 0.004289 |
| NBLA00301 | magenta | -0.09295 | 0.534304 | 0.222943 | 0.131991 |
| AMN | magenta | -0.09138 | 0.541289 | -0.24836 | 0.092313 |
| C10orf55 | magenta | 0.09039 | 0.545688 | 0.665836 | 3.27E-07 |
| FHAD1 | magenta | 0.08967 | 0.548907 | 0.480656 | 0.000627 |
| APOC2 | magenta | 0.086607 | 0.562692 | 0.667754 | 2.94E-07 |
| CCL17 | magenta | 0.08451 | 0.572222 | 0.348258 | 0.016446 |
| RBMXL2 | magenta | 0.083455 | 0.577043 | -0.31777 | 0.029507 |
| NRIP3 | magenta | 0.08165 | 0.585339 | 0.667589 | 2.97E-07 |
| LCE1E | magenta | 0.081075 | 0.58799 | 0.376416 | 0.009113 |
| TRPA1 | magenta | -0.08104 | 0.588132 | 0.565263 | 3.48E-05 |
| SULT1C2 | magenta | 0.080002 | 0.592957 | 0.811986 | 4.33E-12 |
| APBA2 | magenta | 0.078174 | 0.601458 | 0.687592 | 9.35E-08 |
| NKAIN4 | magenta | 0.076136 | 0.611001 | 0.352737 | 0.015022 |
| IRS2 | magenta | 0.071369 | 0.633563 | 0.576227 | 2.25E-05 |
| GJB2 | magenta | 0.070576 | 0.637351 | 0.7357 | 3.84E-09 |
| SAMD5 | magenta | 0.068642 | 0.646626 | 0.42953 | 0.002587 |
| C5orf20 | magenta | 0.066615 | 0.656401 | 0.557884 | 4.62E-05 |
| ACSM5 | magenta | 0.065884 | 0.65994 | 0.447425 | 0.001615 |
| MNX1 | magenta | -0.06562 | 0.661215 | -0.41 | 0.004208 |
| TSPAN10 | magenta | -0.06218 | 0.677993 | 0.320407 | 0.028114 |
| CCDC116 | magenta | -0.05952 | 0.691071 | 0.258906 | 0.078871 |
| SQRDL | magenta | 0.058954 | 0.693857 | 0.539715 | 9.04E-05 |
| ZNF214 | magenta | -0.05698 | 0.703627 | -0.3241 | 0.026251 |
| MEST | magenta | 0.054869 | 0.714136 | -0.42084 | 0.003224 |
| NEURL3 | magenta | 0.052098 | 0.728 | 0.686237 | 1.01E-07 |
| BACH2 | magenta | -0.05077 | 0.734687 | -0.54212 | 8.29E-05 |
| KCNMA1 | magenta | 0.049398 | 0.741598 | 0.628901 | 2.20E-06 |
| GFAP | magenta | -0.04833 | 0.747014 | 0.346234 | 0.017125 |
| TFCP2L1 | magenta | 0.047058 | 0.753444 | 0.266732 | 0.069928 |
| FAM189A1 | magenta | -0.0451 | 0.763403 | 0.378177 | 0.008768 |
| ADAMDEC1 | magenta | 0.044787 | 0.764995 | 0.79934 | 1.62E-11 |
| CACNG4 | magenta | 0.044104 | 0.768479 | 0.652623 | 6.66E-07 |
| USP43 | magenta | 0.042738 | 0.77546 | 0.628466 | 2.25E-06 |
| SLC4A11 | magenta | 0.041724 | 0.780654 | 0.337368 | 0.020393 |
| SPATS1 | magenta | -0.03972 | 0.790926 | 0.454249 | 0.00134 |
| CYP2S1 | magenta | 0.038843 | 0.795466 | 0.795282 | 2.43E-11 |
| DFNA5 | magenta | 0.034529 | 0.817769 | 0.280921 | 0.05578 |
| MYO1A | magenta | -0.03411 | 0.819937 | 0.675908 | 1.85E-07 |
| CES4 | magenta | 0.032432 | 0.828668 | 0.6454 | 9.69E-07 |
| COLEC12 | magenta | 0.031418 | 0.833945 | 0.191337 | 0.197622 |
| SHISA9 | magenta | -0.03109 | 0.835668 | 0.302989 | 0.038434 |
| DGAT2 | magenta | 0.027758 | 0.853061 | 0.544633 | 7.56E-05 |
| KLHL14 | magenta | -0.02721 | 0.855916 | -0.58289 | 1.71E-05 |
| GNAZ | magenta | -0.02656 | 0.859342 | -0.53794 | 9.63E-05 |
| LOC84856 | magenta | -0.02642 | 0.860079 | 0.417962 | 0.003463 |
| SCUBE2 | magenta | -0.02561 | 0.864339 | 0.343017 | 0.018256 |
| PRDM15 | magenta | 0.024081 | 0.872357 | -0.42271 | 0.003076 |
| GABRA5 | magenta | 0.022991 | 0.878088 | 0.361034 | 0.01266 |
| C11orf70 | magenta | 0.022623 | 0.880026 | 0.4937 | 0.000421 |
| SAMD7 | magenta | -0.02164 | 0.885208 | -0.35972 | 0.013012 |
| C22orf34 | magenta | 0.019464 | 0.896678 | -0.46256 | 0.001062 |
| DPYS | magenta | 0.018937 | 0.899461 | 0.58149 | 1.82E-05 |
| MOG | magenta | 0.017898 | 0.904953 | 0.29162 | 0.046715 |
| FRMD5 | magenta | -0.0165 | 0.912341 | -0.25606 | 0.082342 |
| PADI2 | magenta | 0.016383 | 0.912966 | 0.509381 | 0.000255 |
| IL12B | magenta | 0.015863 | 0.915716 | 0.348971 | 0.016212 |
| MGST1 | magenta | 0.012672 | 0.932629 | 0.369647 | 0.010551 |
| ARL4D | magenta | -0.00924 | 0.950828 | 0.389139 | 0.006863 |
| SLIT1 | magenta | -0.00868 | 0.953845 | 0.277577 | 0.058887 |
| KLF14 | magenta | 0.007319 | 0.96106 | -0.3343 | 0.02164 |
| COL9A3 | magenta | -0.00681 | 0.96376 | -0.30916 | 0.034472 |
| HS3ST2 | magenta | -0.00657 | 0.965052 | 0.511337 | 0.00024 |
| CNIH3 | magenta | -0.00492 | 0.973808 | 0.337664 | 0.020276 |
| MEGF11 | magenta | -0.00274 | 0.985418 | 0.440933 | 0.001921 |
| GFRA3 | magenta | 0.001502 | 0.992003 | -0.34805 | 0.016513 |
| CASQ1 | magenta | 0.001 | 0.994675 | 0.659617 | 4.59E-07 |
| MAOB | magenta | -0.00046 | 0.997569 | 0.55314 | 5.52E-05 |
| SLC5A12 | magenta | 0.000441 | 0.997654 | -0.2399 | 0.104334 |
