# Supplementary material for: Identification of PLA2G7 as a novel biomarker of diffuse large B cell lymphoma
Source: BMC Cancer. 2021 Aug 17;21:927. doi: 10.1186/s12885-021-08660-4 (PMC8369790; doi:10.1186/s12885-021-08660-4)
Supplement: Supplementary file 2 — Additional file 2. [file 12885_2021_8660_MOESM2_ESM.docx]

Supplemental Table S2

|  |  |  |  |
| --- | --- | --- | --- |
| Characteristics | PLA2G7 expression | |  |
|  | High,no.cases(>14.24) | Low,no.cases(<=14.24) |  |
| Age(years) |  |  |  |
| ≤52 | 4 | 6 |  |
| >52 | 5 | 3 |  |
| Gender |  |  |  |
| Female | 3 | 4 |  |
| Male | 6 | 5 |  |
| stage |  |  |  |
| I–II | 3 | 2 |  |
| III–IV | 6 | 7 |  |
| Subtype |  |  |  |
| GCB | 7 | 4 |  |
| Non-GCB | 2 | 5 |  |
| IPI score |  |  |  |
| 0-2 | 7 | 7 |  |
| >3 | 2 | 2 |  |
| CR after first-line chemotheray |  |  |  |
| yes | 6 | 7 |  |
| no | 3 | 2 |  |
| serum B2m |  |  |  |
| High | 7 | 2 |  |
| Normal | 2 | 7 |  |
| serum LDH |  |  |  |
| High | 5 | 2 |  |
| Normal | 4 | 7 |  |
| A/B [symptom](C:/Program%20Files%20(x86)/Youdao/Dict/8.9.6.0/resultui/html/index.html#/javascript:;)s |  |  |  |
| A | 2 | 2 |  |
| B | 7 | 7 |  |
| Low serum albumin |  |  |  |
| yes | 2 | 3 |  |
| no | 7 | 6 |  |
| CD10 |  |  |  |
| [positive](C:/Program%20Files%20(x86)/Youdao/Dict/8.9.6.0/resultui/html/index.html#/javascript:;) | 7 | 3 |  |
| [negative](C:/Program%20Files%20(x86)/Youdao/Dict/8.9.6.0/resultui/html/index.html#/javascript:;) | 2 | 6 |  |
| CD20 |  |  |  |
| [positive](C:/Program%20Files%20(x86)/Youdao/Dict/8.9.6.0/resultui/html/index.html#/javascript:;) | 9 | 9 |  |
| [negative](C:/Program%20Files%20(x86)/Youdao/Dict/8.9.6.0/resultui/html/index.html#/javascript:;) | 0 | 0 |  |
| BCL-2 |  |  |  |
| [positive](C:/Program%20Files%20(x86)/Youdao/Dict/8.9.6.0/resultui/html/index.html#/javascript:;) | 7 | 8 |  |
| [negative](C:/Program%20Files%20(x86)/Youdao/Dict/8.9.6.0/resultui/html/index.html#/javascript:;) | 2 | 1 |  |
| BCL-6 |  |  |  |
| [positive](C:/Program%20Files%20(x86)/Youdao/Dict/8.9.6.0/resultui/html/index.html#/javascript:;) | 8 | 7 |  |
| [negative](C:/Program%20Files%20(x86)/Youdao/Dict/8.9.6.0/resultui/html/index.html#/javascript:;) | 1 | 2 |  |

Table S2 Clinicopathological characteristics of DLBCL tissue samples. (GCB: germinal centre B-cell like; IPI: international prognostic index; CR: complete response; B2m: Recombinant Human beta-2-Microglobulin; LDH: lactate dehydrogenase; BCL: B-cell lymphoma.)
